# Perceived algorithmic control and anti-algorithm behaviors: the catalytic role of perceived overqualification

**DOI:** 10.3389/fpsyg.2026.1774556

**Published:** 2026-09-10

**Authors:** Chaoyang Li, Qian Xing

**Affiliations:** 1 Weifang University of Science and Technology, Shouguang, China; 2 Zibo Polytechnic University, Zibo, China

**Keywords:** anti-algorithm behavior, conservation of resources theory, gig economy, perceived algorithmic control, perceived overqualification

## Abstract

**Introduction:**

The rapid expansion of algorithmic management in the gig economy has sparked widespread concerns over workers’ behavioral resistance to algorithmic constraints, yet the underlying psychological mechanism between perceived algorithmic control and workers’ anti-algorithm behavior remains underexplored. This study examines the mechanism linking perceived algorithmic control to workers’ anti-algorithm behavior in the gig economy, with particular attention to the mediating role of perceived overqualification.

**Methods:**

A three-wave longitudinal design was adopted to collect valid data from 483 food delivery riders across three cities in eastern China, and partial least squares structural equation modeling (PLS-SEM) was employed for empirical analysis.

**Results:**

The findings indicate that perceived algorithmic control — operationalized through stringent normative guidance, real-time surveillance, and behavioral constraints — is positively associated with anti-algorithm behavior. Moreover, perceived algorithmic control is positively related to workers’ sense of overqualification by triggering the perceived mismatch between their capabilities and task demands, which in turn correlates with anti-algorithm behavior. Perceived overqualification significantly mediates the control–resistance link, revealing a clear path that algorithmic management practices reshape workers’ overqualification perceptions, and further induce their coping behaviors against algorithmic constraints.

**Discussion:**

These findings illuminate the psychological experience and adaptive process of gig workers when facing algorithmic governance, filling the research gap in the internal transmission mechanism between digital labor management and individual resistance. It also offers targeted theoretical and practical implications for optimizing digital labor regulation and building more harmonious labor relations in the platform economy.

## 1 Introduction

The gig economy has witnessed a profound convergence with digital technology in recent years. Online labor platforms leverage algorithmic systems to efficiently match labor supply with demand, catalyzing the rapid ascent of the platform economy (Kuhn and Maleki, 2017). Today, a substantial proportion of gig workers undertake tasks through internet-based platforms that not only provide employment opportunities but also orchestrate task execution and ensure compensation (Duggan et al., 2020). Platform workers are increasingly functioning as mobile carriers of value within urban landscapes, reshaping the spatial logic of cities (Newlands, 2020).

Within this employment model—characterized by minimal human intervention—online platforms innovatively deploy algorithms to manage geographically dispersed workers operating across temporal and spatial boundaries, extending control beyond the reach of conventional organizational oversight. The academic literature terms this practice “algorithmic control,” defined as workers’ comprehensive perception of digital control mechanisms enacted by algorithmic systems through normative guidance, real-time surveillance, and behavioral constraints (Pei et al., 2021). Specifically, platform enterprises restructure labor processes via a three-dimensional control architecture: normative guidance systems establish service standards and operational protocols, tracking and evaluation systems enable dynamic performance monitoring, and behavioral constraint mechanisms institutionalize reward-and-punishment rule systems. This panoptic form of labor supervision—the algorithmic panopticon—fully embeds gig workers’ labor processes, income distribution, and occupational evaluations within algorithmic logic. However, as perceived algorithmic control becomes structurally embedded in organizational management and its governance boundaries grow increasingly blurred, its deleterious effects on workers have surfaced. Owing to the comprehensiveness, immediacy, interactivity, and opacity of algorithms, workers under perceived algorithmic control face intensified monitoring and constraints, with their work autonomy substantially diminished (Wood et al., 2019). This engenders negative affect such as insecurity, perceived injustice, stress, social alienation, and anxiety (Newman et al., 2020). The mechanistic operational logic of algorithms, insensitive to individual contexts and emotions, not only amplifies surveillance density in the workplace but also positively predicts workers’ experience of profound alienation (Ni and Liu, 2025).

In an era defined by the reshaping of labor value and algorithmic empowerment, an enterprise’s core competitiveness is inextricably linked to workers’ efficiency and service quality (Wu and Li, 2018). While perceived algorithmic control can enhance operational efficiency, it may simultaneously undermine service quality by provoking adverse psychological reactions among workers. Against this backdrop, within algorithm-driven organizational forms, when perceived algorithmic control persistently elicits negative psychological responses, individuals may resort to informal, non-institutionalized covert resistance strategies—a distinctive form of defiance that scholars have termed “Anti-Algorithm Behaviors” (Wei et al., 2024). Accordingly, elucidating workers’ algorithmic perception mechanisms and their coping strategies has become a matter of increasing urgency (Chen, 2020).

A critical yet underexplored factor linking perceived algorithmic control to Anti-Algorithm Behaviors is perceived overqualification. As a subjective individual perception, perceived overqualification denotes the perceived mismatch between one’s qualifications (e.g., education, knowledge, skills) and job demands (McKee-Ryan and Harvey, 2011). Within algorithmic work contexts, gig workers may perceive their extensive capabilities and experiential knowledge as underutilized under the rigid standards and procedures imposed by algorithms, thereby cultivating a sense of overqualification. Workers with high perceived overqualification typically hold the conviction that their skills and competencies are not being fully leveraged. Generally, when individuals perceive that their valuable resources remain underutilized—precluding resource appreciation and commensurate returns—they develop a strong impetus to alter the status quo, seeking to escape the constraints of their current work configuration and secure greater resource-based rewards, such as job satisfaction and career advancement.

The intensification of work rhythms—exemplified by prevalent extreme work schedules in certain gig contexts—demands that workers complete an escalating volume of tasks within increasingly compressed timeframes. Under the intensive and intelligent surveillance of algorithms, workers must invest substantial time and energy to fulfill their obligations, yet confront immense pressure and uncertainty. This high-intensity work investment yields diminishing resource returns, instead precipitating gradual work exhaustion, emotional depletion, and even feelings of incompetence (Cheng et al., 2024). Under such conditions, the proactive aspiration to improve work conditions—originally motivated by perceived overqualification—becomes difficult to realize through legitimate channels, given the high-pressure, restrictive, and inflexible work environment engendered by perceived algorithmic control. When workers perceive that their capabilities are severely constrained by algorithms, their legitimate demands go unaddressed, and their efforts to improve the situation prove futile, they may turn to Anti-Algorithm Behavior as a covert resistance strategy to express dissatisfaction and defiance. They recognize that compliance with algorithms’ predefined rules and procedures perpetuates the suppression of their capabilities, rendering Anti-Algorithm Behavior a seemingly viable means to break free from this dilemma and reclaim work autonomy and recognition of their competencies.

Against this backdrop, the present study, situated within the gig economy and anchored in online labor platforms, constructs a theoretical model—"perceived algorithmic control → perceived overqualification → Anti-Algorithm Behavior”—grounded in Conservation of Resources (COR) theory. It aims to elucidate the mechanism underlying the relationship between perceived algorithmic control and Anti-Algorithm Behavior, revealing how perceived algorithmic control is statistically related to workers’ Anti-Algorithm Behavior. The study seeks to offer theoretically grounded and practically relevant insights for research and practice in this domain.

## 2 Theoretical foundation and hypothesis development

### 2.1 Conservation of resources (COR) theory

Conservation of Resources (COR) theory serves as a foundational framework for understanding the motivational mechanisms underlying employees’ work attitudes and behaviors (Hobfoll, 1989; Westman et al., 2005). The theory posits that individuals are inherently motivated to acquire, retain, foster, and protect resources they value, as these resources are instrumental in adapting to environmental demands and sustaining wellbeing. A central tenet of COR theory is that individuals possessing abundant resources are less vulnerable to resource loss and better positioned to accumulate additional resources. Building on this premise, the theory delineates two critical dynamics: the loss spiral and the gain spiral.

The loss spiral describes a process whereby resource-deficient individuals become increasingly susceptible to stress-induced resource depletion, as their resource investments yield insufficient returns, thereby accelerating further loss (Hobfoll, 2001). In the gig economy, perceived algorithmic control—manifested through opaque task allocation, rigid time constraints, and algorithmic performance evaluation—can erode workers’ autonomy, temporal resources, and psychological energy. When gig workers perceive algorithmic control as excessively stringent or illegitimate, they experience a mismatch between their capabilities and the demands imposed by algorithms. For instance, algorithms may assign tasks incongruent with workers’ skills or preferences, compelling them to invest disproportionate time and effort to comply, thereby depleting their resource reserves.

Conversely, the gain spiral characterizes how resource-sufficient individuals are better equipped to acquire additional resources, generating cumulative resource growth (Hobfoll, 2001). Together, these spirals offer a critical theoretical lens for examining the interplay among perceived algorithmic control, perceived overqualification, and Anti-Algorithm Behaviors. Specifically, unreasonable perceived algorithmic control can initiate a loss spiral: by forcibly assigning skill-mismatched tasks, imposing inflexible deadlines, or applying one-dimensional evaluation criteria, algorithms deplete gig workers’ time and energy resources. This resource depletion cultivates perceived overqualification, as workers come to view their capabilities as constrained and their value as systematically underappreciated. Given the pervasive and opaque nature of algorithmic governance, workers may find it exceedingly difficult to alter the status quo or safeguard their resources through conventional channels, potentially trapping them in a self-reinforcing cycle of sustained resource loss.

In contrast, reasonable algorithmic control can catalyze a gain spiral. When algorithms allocate tasks commensurate with workers’ competencies, provide constructive feedback, and offer development resources, they facilitate resource accumulation and capability enhancement. This alignment between qualifications and job demands attenuates perceived overqualification. Nevertheless, when gig workers encounter resource-related dilemmas that resist resolution through legitimate means, they may resort to Anti-Algorithm Behaviors—a covert resistance strategy—to protect their residual resources and restore resource equilibrium. Such behaviors may include deliberately violating algorithmic rules, strategically delaying task completion, or exploiting algorithmic loopholes, all aimed at reasserting work autonomy and securing recognition for their capabilities.

### 2.2 The relationship between perceived algorithmic control and anti-algorithm behaviors

Against the backdrop of the rapid development of the platform economy, the relationship between gig workers’ perceived algorithmic control and anti-algorithm behavior is a central issue for understanding their work experiences and behavioral evolution. Gig workers’ perception of algorithmic management intensity directly shapes their work stress levels and behavioral tendencies. A higher intensity of perceived algorithmic control increases the likelihood of workers experiencing constraints induced by algorithmic panopticon-like surveillance. Ubiquitous and continuous monitoring keeps them in a prolonged state of tension, with work stress accumulating as management intensity increases. Under such circumstances, to regain work autonomy and alleviate management pressure, workers are more inclined to adopt anti-algorithm behavior, such as exploiting system loopholes and adjusting order-acceptance strategies. Although these behaviors may violate platform rules, they serve as autonomous coping strategies for workers under algorithmic management pressure.

Conservation of Resources (COR) theory provides a rigorous theoretical perspective for elucidating the relationship between the two. The theory posits that resources encompass objects, personal characteristics, conditions, or energies that individuals value, and individuals possess an inherent motivation to protect existing resources and acquire new ones. Changes in individual resources triggered by work events and behaviors directly affect their work perceptions and behavioral outcomes (Hobfoll, 1989). In the context of the platform economy, a sense of control and autonomy are core personal resources highly valued by gig workers. While perceived algorithmic control enhances operational efficiency, it continuously intrudes upon and depletes these core resources. The rigid work norms and process constraints imposed by algorithmic management constantly erode workers’ sense of control and autonomy; the higher the management intensity, the more severe the resource depletion (Halbesleben and Wheeler, 2015).

When employees adopt coping behaviors to ameliorate adverse work situations and enhance their sense of autonomy and efficacy, their resource reservoirs may be expanded (Koopman et al., 2016). However, the comprehensive surveillance under algorithmic management deprives workers of their autonomy. A higher management intensity leads to a more pronounced accumulation of work stress, tension, and anxiety (Petriglieri et al., 2019). Under these circumstances, workers possess a stronger motivation to engage in resistance behaviors to preserve existing resources, replenish depleted personal resources, and mitigate the negative impacts of perceived algorithmic control. Such resistance serves both as an instinctive response to resource depletion and a strategic choice for workers to safeguard their rights and interests within a complex work environment.

From a complementary perspective, although the platform’s perceived algorithmic management system establishes highly standardized performance objectives, it exhibits significant deficiencies in regulating the pathways to achieve these goals—a phenomenon characterized as the “goal-means dualistic rupture.” This creates a structural dilemma for gig workers: the higher the target requirements set by the algorithm, the more limited the legitimate pathways available for workers to meet them. Within this dilemma, to fulfill algorithmic requirements, workers are more inclined to bypass conventional norms and adopt non-compliant methods (Li et al., 2024), thereby precipitating anti-algorithm behavior. Perceived algorithmic control further exacerbates workers’ burdens through refined calculation. Relying on real-time monitoring technologies, it imposes rigid assessments and constraints on the work process; the higher the management intensity, the more protracted the workers’ state of tension. Stringent requirements trigger continuous stress responses, compelling workers to extend their working hours, which may even lead to physiological resource depletion due to overwork (Zhan et al., 2023).

Furthermore, perceived algorithmic control exacerbates the power asymmetry in platform employment relationships. Driven by an algorithm-dominated employment logic, platform workers are subjected to asymmetric evaluation rules and prolonged heavy work demands (Bao et al., 2024). A higher management intensity is associated with continuous stress and the perception of unfairness, relating to more severe psychological resource depletion. The dual depletion of physiological and psychological resources substantially undermines workers’ behavioral self-regulation capacity, further elevating the probability of anti-algorithm behavior. Workers attempt to use these behaviors to circumvent algorithmic constraints, alleviate physical and mental stress, and achieve a relatively equitable working status.

In the platform economy, where algorithmic management predominates, algorithms have become the core mechanism for platforms to allocate resources and execute human resource management practices. Workplaces governed by algorithmic control are no longer traditional labor settings but have evolved into what Kellogg et al. (2020) describe as a “contested terrain of control.” Within this terrain, platforms leverage algorithmic advantages to manage workers, aiming to achieve efficiency and profitability objectives. However, workers are not passive recipients of management; in the face of continuously intensifying algorithmic management, they inevitably adopt resistance measures or proactive strategies to safeguard their rights and interests. This is a fundamental manifestation of human nature and an inevitable outcome of interest bargaining in a market economy (Chen, 2020). Within the platform economy context, such resistance manifests in diverse forms, with anti-algorithm behavior being the most typical. Through anti-algorithm behavior, workers express dissatisfaction and assert their demands, striving to secure greater autonomy and developmental space within the framework of algorithmic management. Furthermore, the higher the management intensity, the more pronounced the tendency to engage in such coping behaviors. Based on the foregoing analysis, we propose the following hypothesis:

*H1*: Perceived algorithmic control positively predicts riders’ anti-algorithm behavior.

### 2.3 The mediating role of perceived overqualification

Perceived overqualification refers to the cognitive state in which employees subjectively assess their knowledge, skills, experience, and comprehensive abilities as significantly exceeding the actual requirements of their current positions (Maynard et al., 2006). At its core, it is a self-evaluation of “person-job mismatch,” fundamentally reflecting the perceived gap between individual capability supply and job capability demand (Wang, 2012). This construct focuses on the cognitive judgment of underutilized abilities, distinguishing it from stress-related experiences such as work overload or emotional exhaustion. When individuals perceive that their abilities cannot be fully utilized in their roles, their positive self-image—established on “ability-role fit”—is undermined (Erdogan and Bauer, 2009). To restore their perception of self-worth and seek avenues for capability utilization, individuals are more prone to adopting deviant behavioral strategies as a coping mechanism (Chen and Liang, 2017).

For food delivery riders, the qualifications and abilities underutilized by their positions encompass several dimensions: first, vocational skills, including route planning, emergency response to unexpected incidents, customer communication and conflict coordination, and multitasking and time management; second, accumulated experience, including judgment regarding the distribution of regional commercial zones and road condition timeliness, as well as general professional literacy and problem-solving skills acquired from previous occupational experiences; third, personal development, including cognitive abilities shaped by formal education and the developmental potential corresponding to career growth aspirations.

Perceived algorithmic control reshapes riders’ assessments of job capability requirements through highly standardized and instruction-driven work processes, thereby altering their perception of person-job fit. To maximize operational efficiency, algorithms decompose the delivery work—which originally required riders’ autonomous decision-making—into a series of linear execution instructions through mechanisms such as smart order dispatching, real-time route planning, and standardized performance feedback. Riders are no longer required to autonomously plan routes, prioritize orders, or flexibly handle delivery exceptions; they need only strictly follow algorithmic instructions to complete their tasks. While enhancing efficiency, this management mode substantially compresses the space for riders’ autonomous decision-making and capability utilization. Consequently, the job’s requirements for comprehensive abilities are significantly weakened, retaining only basic executional requirements. Under these circumstances, riders clearly perceive that their accumulated abilities—such as route planning, communication and coordination, and emergency response—cannot be applied in their work. They realize that their comprehensive qualifications far exceed the job capability requirements defined by the algorithm, thereby forming perceived overqualification.

From the perspective of Conservation of Resources (COR) theory, the core of this process is the continuous depletion of three key resources: work autonomy, space for skill utilization, and professional value recognition. Perceived overqualification is the cognitive evaluation outcome triggered by this resource depletion, rather than the resource itself. Specifically, the rigid management and panopticon-like surveillance of algorithms continuously deplete riders’ work autonomy resources, causing them to lose control over the work process. The instruction-driven nature of workflows constricts the space for capability application, depleting riders’ resources for skill utilization. Meanwhile, standardized performance evaluations focus solely on timeliness and compliance, neglecting the comprehensive ability inputs of riders, which further depletes their resources for professional recognition and sense of value. According to the resource depletion principle of COR theory, when core resources continuously drain without replenishment, individuals initiate a cognitive attribution process to explain the source of the loss. At this point, riders attribute the resource depletion state—characterized by an inability to apply their skills and a lack of recognized value—to a person-job mismatch, specifically believing their qualifications far exceed job requirements. This ultimately shapes the cognitive judgment of perceived overqualification. Compared to emotional experiences such as thwarted autonomy, perceived unfairness, or emotional exhaustion, perceived overqualification is a specific cognitive evaluation targeting the “ability-job” fit. It represents an attributional interpretation of the resource depletion state and serves as a crucial cognitive antecedent for subsequent coping behaviors.

*H2*: Perceived algorithmic control positively predicts perceived overqualification.

Building on the foregoing reasoning, the algorithmic platform functions as the most proximate virtual “leader” with which gig workers interact (Guo and Guo, 2024), exerting a significant influence on the development of perceived overqualification. The degree of leader empowerment delineates the scope of employees’ freedom to act, providing opportunities to demonstrate their competencies and pursue career advancement. When leaders monopolize power, subordinates are deprived of training opportunities, their creativity is suppressed, and they fail to derive meaning and a sense of control from their work—conditions that readily cultivate perceived overqualification (Li and Li, 2021).

From the perspective of Conservation of Resources (COR) theory, individuals possess finite resource pools and are more strongly motivated to protect existing resources than to acquire new ones. When individuals perceive that their resources are being continuously depleted without commensurate returns, they are driven to take immediate action to arrest further loss and avert a self-reinforcing loss spiral (Ding et al., 2019). Overqualified employees, by virtue of their elevated competencies, experience, and resource endowments, harbor heightened expectations for self-actualization and resource utilization in the workplace. However, perceived algorithmic control may impede their capacity to protect and leverage these resources. According to Zhan et al. (2023), algorithms impose top-down control over platform workers through stringent surveillance and rigorous evaluation, substantially attenuating their voice and initiative, exacerbating their sense of relative deprivation, and potentially relating to negative affect and destructive behaviors. Overqualified employees, owing to their superior capabilities, hold particularly strong demands for autonomy and voice. The stringent algorithmic control renders their advantages underutilized, intensifying their sense of relative deprivation and rendering them more susceptible to adopting Anti-Algorithm Behaviors as a resource-protective strategy.

Shen (2022) further contends that perceived algorithmic control resists standardization in flexible, dynamic work contexts, compelling platform workers to expend substantial resources to counteract work-related uncertainties and risks—a process that is positively associated with perceived injustice and resistance. Overqualified employees typically possess clearer career plans and more discerning professional judgments. When algorithmic control constrains their capabilities through rigid rules, their sense of injustice is amplified, making them more inclined to express dissatisfaction and contest algorithmic authority through Anti-Algorithm Behaviors.

Within the norm-governed labor service process, algorithmic rules are oriented toward satisfying customer demands, yet tasks are assigned stochastically based on these demands. To secure a stable income, gig workers must remain online virtually around the clock (Liu et al., 2022). This irregular, high-intensity work regime escalates workload and work pressure (Wood et al., 2019). For overqualified gig workers, who initially anticipate that their work will align with their capabilities—enabling effective resource utilization and career development—the high-load, low-match work model imposed by algorithms signals resource wastage. To safeguard their resources and reclaim work equity, they may resort to Anti-Algorithm Behaviors, such as declining unreasonable task assignments and contesting algorithmic rules.

Based on the foregoing analysis, we propose the following hypotheses:

*H3*: Perceived overqualification positively predicts riders’ anti-algorithm behavior.

*H4*: Perceived overqualification plays a mediating role in the temporal association between perceived algorithmic control and anti-algorithm behavior.

## 3 Data sources and variable selection

### 3.1 Sample and data sources

To mitigate common method bias, this study employed a three-wave longitudinal design with a one-week interval between adjacent surveys. This setup aligns with the high mobility characteristic of food delivery riders and temporally separates variable measurements to reduce the interference of common method variance.

Cross-period questionnaire matching adopted a joint coding scheme comprising the “last four digits of the mobile phone number + two stable baseline attributes.” The last four digits of the respondent’s mobile phone number served as the core tracking identifier, while two fixed attributes collected during the baseline survey—located city and employment type—were matched as auxiliary verification identifiers, jointly constituting a unique matching key. These two attributes were selected as auxiliary identifiers because they are stable characteristics that do not change across survey waves, effectively improving matching accuracy without collecting additional sensitive information. Prior to formal matching, this study conducted a preliminary validation of coding uniqueness using the 508 valid samples from Wave 1. When using only the last four digits of the mobile phone number, 2 sets of duplicate codes appeared (each set containing 2 respondents, totaling 4 individuals). After incorporating the two auxiliary identifiers (city and employment type), the joint codes for all samples were uniquely distinguishable within the scope of this study, with no indistinguishable duplicate cases. The matching work for all three waves of questionnaires was completed based on this joint coding. No ambiguous matching situations where identities could not be confirmed occurred throughout the process, and no valid samples were excluded due to matching accuracy issues. Ultimately, a total of 483 valid matched samples completed all three waves of the survey, with sample attrition solely caused by respondents’ non-participation in subsequent surveys.

This study strictly adhered to research ethics. The complete mobile phone numbers provided by respondents were used for only two purposes: extracting the last four digits to generate tracking codes, and distributing survey incentives. The complete mobile phone numbers and questionnaire response data were managed using separated storage and independent encryption, accessible only to designated researchers. Upon completion of the three-wave questionnaire matching and the distribution of survey incentives, all complete mobile phone number information was permanently deleted. All subsequent statistical analyses were conducted using anonymized response data, making it impossible to reverse-identify respondents’ personal identities through the data. Furthermore, this study explicitly committed to the cooperating platform that the platform would not obtain any personal information or individual response content from the respondents, thereby fully ensuring response anonymity and reducing respondents’ concerns regarding sensitive items.

The target population of this study consists of food delivery riders in three cities in eastern China: Shanghai, Hangzhou, and Suzhou. In collaboration with Meituan, a mainstream local delivery platform, data collection was conducted using convenience sampling. With the platform’s assistance, respondents were recruited through offline rider stations and online rider communities. The inclusion criteria were riders currently on duty with at least 3 months of work experience, ensuring that respondents had sufficient exposure to and perception of the platform’s algorithmic management practices. Respondents who completed all three waves of the survey received a 15 RMB WeChat red packet as a cash reward.

This study employed a three-wave longitudinal design. Data collection was conducted in Shanghai, Hangzhou, and Suzhou from June 2025 to October 2025. Respondents were recruited through both offline rider stations and online rider communities, with a one-week interval set between adjacent survey waves. Wave 1 measured perceived algorithmic control, demographic variables, and control variables, distributing 532 questionnaires and collecting 508 valid responses. Wave 2 collected data on perceived overqualification from the 508 tracked respondents, yielding 497 valid responses. Wave 3 measured anti-algorithm behaviors among the 497 tracked respondents, resulting in 483 valid responses.

This study uniformly established two criteria for questionnaire validity: first, questionnaires with a missing rate exceeding 10% on core research variable items, or lacking the tracking codes used for cross-period matching, were excluded from the valid sample scope; second, samples exhibiting obvious logical contradictions across the three waves of tracking responses were excluded from the final analysis dataset.

Regarding the distribution of sample stations, respondents recruited through offline channels were dispersed across 32 independent rider stations in the three cities. The final valid sample size per station did not exceed 25 individuals, preventing a high concentration of samples in any single station. The data does not exhibit strong station clustering characteristics and will not systematically interfere with the core model estimation, precluding the need for additional multilevel linear modeling for cluster correction.

To examine whether systematic bias existed in sample attrition during the tracking process, this study utilized Wave 1 baseline data to compare the baseline characteristics of respondents who completed all three waves of the survey with those who dropped out midway. Chi-square tests were employed for categorical demographic variables, and independent-samples t-tests were used for the continuous variable of perceived algorithmic control.

The results indicated no significant differences between respondents who completed the entire survey and those who dropped out in terms of gender (*χ*^2^ = 2.675, df = 1, *p* = 0.102), age (*χ*^2^ = 1.279, df = 4, *p* = 0.865), education (*χ*^2^ = 1.903, df = 4, *p* = 0.754), employment type (*χ*^2^ = 0.082, df = 1, *p* = 0.775), or years of experience (*χ*^2^ = 1.810, df = 3, *p* = 0.613). Regarding the level of perceived algorithmic control, the completion group (*N* = 483, *M* = 3.89, SD = 0.82) and the attrition group (*N* = 25, *M* = 3.86, SD = 0.74) similarly showed no significant difference (*t* = 0.168, df = 506, *p* = 0.866).

The *p*-values for all tests were greater than the 0.05 significance level, detecting no systematic differences between the two groups in the baseline observed variables. This indicates that this study did not experience significant non-random sample attrition, and the overall sample structure remained stable.

The final sample (*N* = 483) comprised 309 males (64.0%) and 174 females (36.0%), reflecting a notable gender imbalance consistent with the demographic composition of the gig delivery workforce. Age was relatively dispersed: 50 respondents (10.4%) were aged 20 or below; 125 (25.9%) were aged 21–29; 115 (23.8%) were aged 30–39; 159 (32.9%) were aged 40–49 (the largest group); and 34 (7.0%) were aged 50 or above. The 40–49 age cohort dominated, while the 21–39 cohort collectively accounted for nearly half the sample. Educational attainment varied considerably: 71 respondents (14.6%) held a junior high school education or below; 166 (34.4%) held a high school diploma; 195 (40.4%) held a junior college degree (the largest group); 37 (7.7%) held a bachelor’s degree; and 14 (2.9%) held a postgraduate degree or above. Junior college and high school graduates constituted the majority, while highly educated respondents were underrepresented. Regarding work experience, 60 respondents (12.4%) had 3 years or less; 197 (40.8%) had 4–6 years; 196 (40.6%) had 7–9 years; and 30 (6.2%) had 10 or more years. The 4–9 year cohort accounted for approximately 80% of the sample, indicating moderate concentration in tenure. Work form was nearly evenly distributed: 237 part-time riders (49.1%) and 246 full-time riders (50.9%), suggesting diverse employment preferences among participants. Regarding city distribution, 142 respondents were from Shanghai (29.4%), 250 from Hangzhou (51.8%), and 91 from Suzhou (18.8%). In terms of recruitment channels, 102 respondents were recruited offline (21.1%), while 381 were recruited online (78.9%).

### 3.2 Variable Measurement

All measurement scales employed in this study were derived from established instruments, whose reliability and validity have been confirmed in prior research. All items were measured on a 5-point Likert scale ranging from 1 (strongly disagree) to 5 (strongly agree).

To ensure cross-cultural applicability, this study adhered to a standard back-translation procedure (Brislin, 1970). First, a bilingual researcher translated the original English scales into Chinese. Subsequently, an independent researcher, blind to the original scales, back-translated the Chinese version into English. The two researchers then collaboratively compared the two versions and reconciled wording discrepancies. Additionally, two senior riders with over 5 years of platform experience were invited to pre-test the items. Overly academic phrasing was replaced with accessible terminology to ensure the items closely aligned with the research context and were easily comprehensible for the riders.

Perceived algorithmic control. The measurement items were adapted from the established scale developed by Pei et al. (2021). The original scale conceptualizes perceived algorithmic control as a multidimensional construct comprising three core dimensions: normative guidance, tracking and evaluation, and behavioral constraint, systematically capturing the governance logic of platform algorithms over work processes, performance, and behavioral boundaries.

Based on the actual work scenarios of food delivery riders and the core objectives of this study, the final items were determined through a systematic screening process. First, an exploratory factor analysis was conducted on 30 pilot samples to retain items with factor loadings above 0.7 and eliminate those with high cross-loadings or semantic ambiguity. Second, three researchers in the field of platform employment and two senior riders were invited to review content validity, evaluating and revising items regarding semantic adaptability and contextual fit. Third, items representing the most intensely perceived and representative algorithmic management practices in riders’ daily work were prioritized, ultimately retaining four core items. It should be noted that this study utilizes a context-adapted abbreviated scale rather than a standardized short form developed by the original authors; its measurement semantics have been contextualized for the food delivery scenario, and no claim is made regarding the complete coverage of all dimensions of the original scale.

This scale contains four items: (1) The algorithm intelligently allocates my work tasks; (2) The algorithm provides me with real-time dynamic feedback on my work performance; (3) The algorithm offers cash rewards during specific time periods to incentivize me to work harder; (4) When my work fails to meet platform requirements, the algorithm imposes fines on me.

Theoretically, these four items can be categorized into two types of algorithmic governance practices. “The algorithm intelligently allocates my work tasks” and “The algorithm provides me with real-time dynamic feedback on my work performance” belong to process standardization practices; the former solidifies work paths through task dispatching rules, while the latter clarifies operational standards through real-time performance information, jointly achieving standardized management of the work process. “The algorithm offers cash rewards during specific time periods to incentivize me to work harder” and “When my work fails to meet platform requirements, the algorithm imposes fines on me” belong to behavioral constraint practices; these directly guide and restrict riders’ behavioral choices through a dual mechanism of positive incentives and negative penalties.

Although theoretical subdivisions are possible, this study conceptualizes perceived algorithmic control as a unidimensional reflective construct, primarily characterizing riders’ overall perception of the management intensity of algorithmic intervention in their work. This specification is supported by both theoretical rationale and statistical evidence. Theoretically, both process standardization and behavioral constraint practices are rooted in the unified systemic logic of platform algorithmic governance, jointly pointing to the core latent variable of “the degree of algorithmic control over work processes and behaviors.” When riders perceive an increase in the overall intensity of algorithmic management, their perceptions across the four practices—task allocation, performance feedback, and reward/penalty mechanisms—increase synchronously. This aligns with the causal assumption of a reflective construct (“construct as cause, items as effect”), making it appropriate to aggregate them into a comprehensive index reflecting the overall perception level. Statistically, the reflective measurement model test based on SmartPLS showed that the outer loadings of the four items ranged from 0.766 to 0.817, all exceeding the 0.7 threshold. The composite reliability (CR) was 0.876, and the average variance extracted (AVE) was 0.639, satisfying convergent validity requirements. Cross-loadings tests further indicated that all items loaded significantly higher on the target construct than on other constructs, indicating no cross-factor confusion.

From a research design perspective, this study aims to examine how food delivery riders’ overall perception of algorithmic control affects their coping behaviors, focusing on revealing the overall effect pathway rather than comparing the differential effects of various control dimensions. Adopting a unidimensional integrated model aligns with the theoretical framework of this study, enhancing model parsimony and explanatory power, and fulfilling the core research objectives.

Perceived overqualification. Adapted from the scale by Chen et al. (2017), perceived overqualification comprises four items: (1) My previous training has not been fully utilized in this job; (2) My educational level exceeds the requirements of my current position; (3) People with less work experience than me are also competent in my job; (4) Some of my work skills are of no use in my current position.

Anti-algorithm behavior. Referring to the study by Wei et al. (2024), anti-algorithm behavior is measured by four items: (1) When approaching the delivery time limit, I click “delivered” in advance after obtaining the customer’s consent; (2) I set a virtual location to receive orders; (3) I only turn on the APP to wait for dispatch after arriving at commercial zones with high foot traffic; (4) I refuse to accept orders from low-rated merchants.

In this study, anti-algorithm behavior is defined as a category of informal, rule-deviating coping strategies adopted by riders in response to perceived algorithmic control. The specific connotations of each item are as follows: Item 1 reflects riders bypassing platform delivery time limit requirements to avoid penalties for delays. Item 2 captures riders evading the algorithm’s location-matching dispatch mechanism. Item 3 refers to riders actively circumventing the algorithm’s long-distance dispatch rules by selectively activating the APP only in high-demand areas. Item 4 denotes riders deliberately refusing orders from low-rated merchants to avoid negative outcomes such as bad reviews or delays, constituting a form of resistance against the algorithm’s order assignment authority.

### 3.3 Descriptive statistical analysis

As shown in Table 1, the means for perceived algorithmic control, perceived overqualification, and anti-algorithm behaviors are 3.89, 3.92, and 4.02, respectively. Overall, these variables are at a moderately high level, which is consistent with the realistic work characteristics of food delivery riders. The standard deviations of the three variables are all approximately 0.81, with values spanning the entire range of 1.25–5.00, indicating sufficient data variance. Regarding distribution shape, all three variables exhibit varying degrees of negative skewness, with absolute skewness values ranging from 1.10 to 1.67. This represents mild to moderate skewness, and no severe ceiling effect is observed. Furthermore, gradient differences exist among the variable means; coupled with the common method bias test results, this rules out the interference of severe acquiescence response bias.

**Table 1:** Descriptive statistical analysis.

Variables	Min	Max	Mean	Std. error	Std. deviation	Skewness		Kurtosis	
						Statistic	Std. error	Statistic	Std. error
Perceived algorithmic control	1.25	5.00	3.8882	0.03730	0.81983	−1.343	0.111	1.113	0.222
Perceived overqualification	1.25	5.00	3.9244	0.03671	0.80679	−1.104	0.111	0.713	0.222
Anti-algorithm behavior	1.25	5.00	4.0160	0.03674	0.80753	−1.667	0.111	2.174	0.222

## 4 Data analysis and results

To ensure the accuracy and reliability of our findings, we employed a suite of statistical procedures to detect and control for potential biases. First, Harman’s single-factor test was conducted using SPSS 27.0 to assess common method bias and verify that the factor structure was not driven by a single source. This step confirms the multifactorial integrity of the data, bolstering the study’s internal validity.

This study utilized SmartPLS 3.0 software to conduct hypothesis testing and model evaluation through partial least squares structural equation modeling (PLS-SEM). The core rationale for selecting this method is as follows:

First, the alignment between research objectives and estimation precision. The core of this study is to test the mediating mechanism at the latent variable level: “perceived algorithmic control → perceived overqualification → anti-algorithm behavior.” Compared to manifest variable regression analysis based on item mean scores, PLS-SEM, as a latent variable modeling method, can effectively control measurement error and yield more accurate path coefficient and effect size estimates. Meanwhile, this method allows for the simultaneous completion of measurement model reliability and validity testing, structural path estimation, mediation effect inference, and multi-dimensional model quality evaluation (e.g., *R*^2^, *f*^2^, *Q*^2^) within a unified analytical framework, enabling a systematic and comprehensive response to the research questions.

SmartPLS implements partial least squares structural equation modeling (PLS-SEM), a robust technique for evaluating theoretically derived models. As Khoi and Tuan (2018) noted, SEM enables the simultaneous estimation of multiple relationships within causal frameworks. A key advantage of PLS-SEM is its distribution-free estimation, which relaxes the assumption of multivariate normality and thus offers greater flexibility in applied settings. Diagnostic results on the distribution of core variables indicate that perceived algorithmic control (skewness = −1.343, kurtosis = 1.113), perceived overqualification (skewness = −1.104, kurtosis = 0.713), and anti-algorithm behavior (skewness = −1.667, kurtosis = 2.174) all exhibit varying degrees of negative skewness and leptokurtic characteristics, failing to satisfy the assumption of multivariate normality. The maximum likelihood estimation of traditional covariance-based structural equation modeling (CB-SEM) imposes strict requirements on normality; non-normal data can easily lead to biased standard errors and distorted model fit indices. In contrast, PLS-SEM employs non-parametric bootstrapping for statistical inference and does not rely on distributional assumptions, demonstrating stronger robustness to the non-normal data in this study and yielding more stable parameter estimation results.

The PLS approach is widely recognized as a versatile analytical tool across econometrics, sociometry, and psychometrics (Guinot et al., 2001), with extensive applications in business strategy, global integration, and organizational research. Together, these methods provide a rigorous analytical framework for examining the relationship between perceived algorithmic control and anti-algorithm behavior, yielding nuanced insights into the underlying mechanisms.

### 4.1 Common method bias test

All data in this study were collected via employee self-reports. Although the anonymous three-wave longitudinal design mitigated common source bias at the procedural level, this risk cannot be completely eliminated. Consequently, this study adopted two statistical approaches for verification.

First, a full collinearity test, which is standard practice in the PLS-SEM domain, was employed as the primary detection method. As shown in Table 2, the variance inflation factor (VIF) values for all latent variables in the model ranged from 1.257 to 1.908, all below the critical threshold of 3.3, indicating that the model is free from severe common method bias.

**Table 2:** VIF values of all latent variables.

Latent variables	VIF values
AAB1	1.805
AAB2	1.618
AAB3	1.519
AAB4	1.719
PAC1	1.908
PAC2	1.627
PAC3	1.702
PAC4	1.557
POQ1	1.424
POQ2	1.459
POQ3	1.257
POQ4	1.546

Second, Harman’s single-factor test was employed for supplementary verification. All 12 measurement items were subjected to an unrotated principal component analysis (PCA). As shown in Table 3, a total of 3 factors with eigenvalues greater than 1 were extracted, with the first factor accounting for 41.46% of the variance, which is below the generally acceptable threshold of 50%. Combining the procedural controls with the results of the two statistical tests, common method bias in this study is within an acceptable range and will not substantially interfere with the core research conclusions.

**Table 3:** Total variance explained.

Component	Initial eigenvalues			Extraction sums of squared loadings			Rotation sums of squared loadings		
	Total	% of variance	Cumulative %	Total	% of variance	Cumulative %	Total	% of variance	Cumulative %
1	4.975	41.461	41.461	4.975	41.461	41.461	2.611	21.758	21.758
2	1.254	10.450	51.912	1.254	10.450	51.912	2.574	21.448	43.206
3	1.224	10.199	62.111	1.224	10.199	62.111	2.269	18.905	62.111
4	0.798	6.653	68.764						
5	0.720	5.998	74.762						
6	0.536	4.464	79.226						
7	0.501	4.175	83.401						
8	0.477	3.975	87.376						
9	0.448	3.737	91.112						
10	0.389	3.244	94.356						
11	0.356	2.964	97.320						
12	0.322	2.680	100.000						

Extraction method: PCA.

KMO = 0.878; *χ*^2^ = 2142.749, df = 66, *p* < 0.001.

### 4.2 Measurement model evaluation

#### 4.2.1 Reliability analysis

Reliability was assessed using three complementary criteria: Cronbach’s alpha, composite reliability (CR), and average variance extracted (AVE). As presented in Table 4, both CR and Cronbach’s alpha for all three constructs—anti-algorithm behavior, perceived algorithmic control, and perceived overqualification—exceeded the conventional threshold of 0.70. Moreover, AVE values for all constructs surpassed 0.50, confirming satisfactory convergent validity and strong internal consistency of the scales.

**Table 4:** Results of reliability test.

Variable	Item	Standardized factor loading	Cronbach’s alpha	CR	AVE
Anti-algorithm behavior	AAB1	0.812	0.801	0.870	0.626
	AAB2	0.766			
	AAB3	0.769			
	AAB4	0.817			
Perceived algorithmic control	PAC1	0.845	0.813	0.876	0.639
	PAC2	0.801			
	PAC3	0.762			
	PAC4	0.788			
Perceived overqualification	POQ1	0.746	0.738	0.835	0.560
	POQ2	0.772			
	POQ3	0.708			
	POQ4	0.764			

With respect to model fit, the results yielded SRMR = 0.081, *χ*^2^ = 445.718, and NFI = 0.794. The SRMR value fell marginally above the recommended cutoff of 0.08 (Henseler et al., 2016), while the NFI was below the conventional 0.90 threshold. These indices suggest that the model does not achieve an ideal fit by covariance-based SEM standards. However, it is well established that PLS-SEM employs distinct evaluation criteria, and the reported values remain within ranges commonly deemed acceptable in the PLS-SEM literature (Hair et al., 2019). Accordingly, the measurement model demonstrates sufficient reliability and validity to support subsequent structural analysis and hypothesis testing.

#### 4.2.2 Validity assessment

Validity concerns the extent to which a measurement instrument captures the construct it intends to measure. In PLS-SEM, discriminant validity is typically evaluated through three complementary approaches: cross-loadings, the Fornell–Larcker criterion, and the heterotrait–monotrait (HTMT) ratio.

First, cross-loadings were examined to ensure that each indicator loaded more strongly on its assigned construct than on any other. As reported in Table 5, all item loadings exceeded 0.70, and each indicator’s loading on its target construct surpassed its cross-loadings on all other constructs, satisfying the discriminant validity requirement (Hair et al., 2019).

**Table 5:** Cross-loading validity test table.

Variables	Anti-algorithm behavior	Perceived algorithmic control	Perceived overqualification
AAB1	**0.812**	0.438	0.358
AAB2	**0.766**	0.389	0.393
AAB3	**0.769**	0.438	0.418
AAB4	**0.817**	0.471	0.395
PAC1	0.462	**0.845**	0.493
PAC2	0.476	**0.801**	0.424
PAC3	0.317	**0.762**	0.334
PAC4	0.474	**0.788**	0.430
POQ1	0.376	0.349	**0.746**
POQ2	0.391	0.447	**0.772**
POQ3	0.382	0.435	**0.708**
POQ4	0.321	0.345	**0.764**

The values in bold in this cross-loading table represent the indicator loadings that exceed 0.7 on their corresponding target latent variables, which indicates that these measurement items have extremely high convergent validity and strong explanatory power for their respective latent constructs.

Second, the Fornell–Larcker criterion requires that the square root of a construct’s AVE exceed its correlations with all other constructs. As shown in Table 6, this condition was met for every construct pair, providing further evidence of discriminant validity.

**Table 6:** Fornell-Larcker criterion test table.

Variables	Anti-algorithm behavior	Perceived algorithmic control	Perceived overqualification
Anti-algorithm behavior	0.791		
Perceived algorithmic control	0.550	0.800	
Perceived overqualification	0.495	0.533	0.748

Third, the HTMT ratio was computed as the ratio of between-construct to within-construct indicator correlations. The conventional threshold of 0.85 was applied, with a relaxed cutoff of 0.90 permissible for conceptually proximate constructs. As presented in Table 7, all HTMT values fell below 0.85, confirming that discriminant validity is not compromised.

**Table 7:** HTMT test table.

Variables	Anti-algorithm behavior	Perceived algorithmic control	Perceived overqualification
Anti-algorithm behavior			
Perceived algorithmic control	0.667		
Perceived overqualification	0.638	0.669	

### 4.3 Structural model evaluation

#### 4.3.1 Multicollinearity test

Multicollinearity was examined using the variance inflation factor (VIF). As reported in Table 8, all VIF values fell well below the conservative threshold of 5.0 (Hair et al., 2019), indicating that multicollinearity does not pose a substantive threat to the estimation of structural path coefficients.

**Table 8:** Results of multicollinearity test for the structural model.

Variables	Anti-algorithm behavior	Perceived algorithmic control	Perceived overqualification
Anti-algorithm behavior			
Perceived algorithmic control	1.397		1
Perceived overqualification	1.397		

#### 4.3.2 Basic model evaluation

This study determined the significance of the hypotheses through standardized path coefficients, and evaluated model quality comprehensively using three complementary metrics: explanatory power (*R*^2^), predictive relevance (*Q*^2^), and effect size (*f*^2^).

In terms of explanatory power, the *R*^2^ value for perceived overqualification is 0.284, and for anti-algorithm behavior, it is 0.359. Both values exceed the 0.25 threshold for weak explanatory power, falling within an acceptable range (Hair et al., 2019).

Regarding predictive relevance, this study employed the Stone–Geisser blindfolding procedure to estimate the *Q*^2^ values. The parameters were set as follows: omission distance *D* = 7, using the cross-validated redundancy approach for calculation. The results showed that the *Q*^2^ value for perceived overqualification was 0.153, and for anti-algorithm behavior, it was 0.219. Both values are greater than 0, indicating that the model possesses acceptable predictive relevance (Hair et al., 2019).

In terms of effect size, the *f*^2^ of perceived algorithmic control on anti-algorithm behaviors was 0.178, achieving a medium effect level; the *f*^2^ of perceived algorithmic control on perceived overqualification was 0.397, reaching a large effect level; and the *f*^2^ of perceived overqualification on anti-algorithm behaviors was 0.089, representing a small to medium effect. All three pathways possess substantial explanatory significance.

#### 4.3.3 Direct effect test

**Figure 1:**
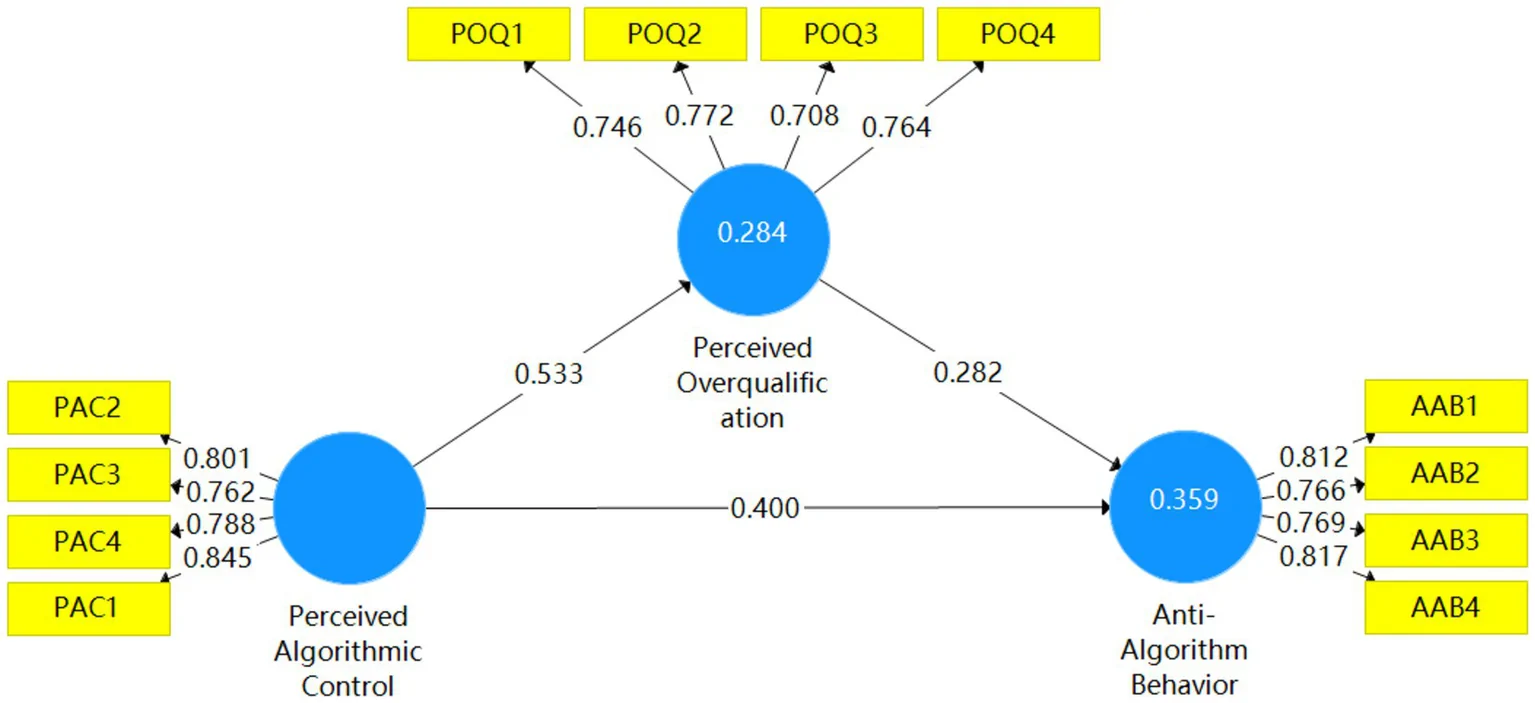
Results of basic model test. Blue circles denote latent variables and yellow rectangles denote observed items. Values on the arrows from latent variables to their corresponding observed items are standardized factor loadings, which reflect the contribution of each item to its affiliated latent variable. Values on the arrows between latent variables are standardized path coefficients, representing the magnitude of direct effects among variables. The values inside endogenous latent variables are the coefficient of determination (*R*^2^), which measures the proportion of variance in the variable explained by the model.

Direct effects were estimated using the bootstrap procedure with 10,000 resamples (Chin, 2009). Results are summarized in Table 9 and Figure 1.

**Table 9:** Path coefficients and their significance.

Paths	Estimated value	Standard deviation	*T*	2.5%	97.5%
Direct effect					
Perceived algorithmic control → Anti-algorithm behavior	0.400	0.048	8.345^***^	0.302	0.497
Perceived algorithmic control → Perceived overqualification	0.533	0.045	11.946^***^	0.437	0.617
Perceived overqualification → Anti-algorithm behavior	0.282	0.049	5.791^***^	0.184	0.375
Mediation effect					
Perceived algorithmic control → Perceived overqualification → Anti-algorithm behavior	0.150	0.028	5.312^***^	0.095	0.206
Total effect					
Perceived algorithmic control → Anti-algorithm behavior	0.550	0.048	11.367^***^	0.445	0.637
Perceived algorithmic control → Perceived overqualification	0.533	0.045	11.946^***^	0.436	0.609
Perceived overqualification → Anti-algorithm behavior	0.282	0.049	5.791^***^	0.186	0.371

Results are based on 10,000 bootstrap resamples using the Bootstrap method.

*p < 0.05, **p < 0.01, ***p < 0.001.

Perceived algorithmic control exerted a significant positive effect on anti-algorithm behavior (*β* = 0.400, *t* = 8.345, *p* < 0.001), supporting H1. The effect of perceived algorithmic control on perceived overqualification was also significant and positive (*β* = 0.533, *t* = 11.946, *p* < 0.001), supporting H2. Moreover, perceived overqualification significantly predicted anti-algorithm behavior (*β* = 0.282, *t* = 5.791, *p* < 0.001), lending support to H3.

Notably, perceived algorithmic control demonstrated the strongest direct effect among all paths (*β* = 0.533), suggesting it is strongly associated with perceived overqualification in this model.

#### 4.3.4 Mediation effect test

To test the mediating role of perceived overqualification, we applied the bootstrap procedure with 10,000 resamples. Results are summarized in Table 6. The indirect effect of perceived algorithmic control on anti-algorithm behavior through perceived overqualification was significant (*β* = 0.150, SD = 0.028, *p* < 0.001), supporting H4.

This indirect effect accounts for approximately 27.3% of the total effect (*β*_total = 0.550), indicating a meaningful mediating mechanism (Hair et al., 2019). The result suggests that perceived overqualification serves as a substantive mediator linking perceived algorithmic control to anti-algorithm behavior, consistent with the theorized pathway.

#### 4.3.5 Measurement model sensitivity analysis

Given that the behavioral boundaries of items (3) “I only turn on the APP to wait for dispatch after arriving at commercial zones with high foot traffic” and (4) “I refuse to accept orders from low-rated merchants” within the anti-algorithm behavior construct are open to some discussion, to rule out the possibility that the core conclusions are driven by individual items, this study conducted a measurement model sensitivity analysis by removing the contested items. Two alternative schemes were designed: Scheme 1 excluded item AAB4, retaining 3 items to measure anti-algorithm behavior; Scheme 2 simultaneously excluded both AAB3 and AAB4, retaining 2 core items. Both models maintained identical settings for all other variables, path structures, and control variables, and the PLS-SEM algorithm and bootstrap testing were re-run.

The test results are presented in Table 10. Overall, the core conclusions of the two robustness models are highly consistent with the baseline model, demonstrating that the research findings possess good robustness, specifically manifested in the following three aspects:

**Table 10:** Robustness check for core path coefficients.

Core paths	Baseline model	Robustness test 1 (AAB4 excluded)	Robustness test 2 (AAB3 and AAB4 excluded)
Perceived algorithmic control → Anti-algorithm behavior	*β* = 0.400, *p* < 0.001	*β* = 0.369, *p* < 0.001	*β* = 0.338, *p* < 0.001
Perceived algorithmic control → Perceived overqualification	*β* = 0.533, *p* < 0.001	*β* = 0.532, *p* < 0.001	*β* = 0.532, *p* < 0.001
Perceived overqualification → Anti-algorithm behavior	*β* = 0.282, *p* < 0.001	*β* = 0.286, *p* < 0.001	*β* = 0.245, *p* < 0.001
Perceived algorithmic control → Perceived overqualification → Anti-algorithm behavior	*β* = 0.150, *p* < 0.001	*β* = 0.152, *p* < 0.001	*β* = 0.130, *p* < 0.001
Anti-algorithm behavior *R*^2^	0.359	0.259	0.328

*p < 0.05, **p < 0.01, ***p < 0.001.

First, the first half of the path is highly stable. The positive effect coefficient of perceived algorithmic control on perceived overqualification remained virtually unchanged across the three models (0.533 → 0.532 → 0.532), showing almost no fluctuation, and all were significant at the *p* < 0.001 level. This indicates that this path is entirely unaffected by the inclusion or exclusion of anti-algorithm behavior measurement items, and the results are highly reliable.

Second, the direction and significance of the core effects remained unchanged. The direct effect of perceived algorithmic control, the positive effect of perceived overqualification, and the mediating indirect effect of perceived overqualification all remained positive and significant (*p* < 0.001) in both robustness models, with no reversal of direction or loss of significance. Specifically, the mediating indirect effect in Scheme 1 (*β* = 0.152) was largely on par with the baseline model (*β* = 0.150), indicating extremely strong stability in the mediation path; although the coefficient in Scheme 2 experienced a slight decline, it remained highly significant. Overall, the coefficient fluctuations were within a reasonable range and did not alter the core underlying patterns.

Third, the model’s explanatory power was maintained at a reasonable level. The *R*^2^ for anti-algorithm behavior was 0.359 in the baseline model, and 0.259 and 0.328, respectively, after item removal. The model still possessed strong explanatory power for the variables, and there was no substantial attenuation in explanatory power due to the reduction in measurement items.

In summary, after removing the items with debatable boundaries, the core effect paths and mediation effects in this study remained stable, ruling out the possibility that the core conclusions were driven by individual measurement items. Both the measurement model and the research conclusions demonstrate good robustness.

#### 4.3.6 Analytical method robustness check

To test the robustness of the conclusions, this study employed the Hayes PROCESS macro (Model 4) to conduct a mediation analysis with 5,000 Bootstrap resamples. The specific results are shown in Tables 11, 12. The regression results indicated that all regression models were overall significant. Perceived algorithmic control positively predicted perceived overqualification (*β* = 0.512, *t* = 13.346, *R*^2^ = 0.270). Without including the mediating variable, the total effect of perceived algorithmic control on anti-algorithm behavior was significant (total effect = 0.534, *t* = 14.157, *R*^2^ = 0.294). When the independent variable and the mediating variable were simultaneously included, both the direct effect of perceived algorithmic control on anti-algorithm behavior (*β* = 0.388, *t* = 9.167) and the predictive effect of perceived overqualification on anti-algorithm behavior (*β* = 0.286, *t* = 6.653) remained significant. The decomposition of the mediation effect revealed that the indirect effect of perceived overqualification was 0.146, with a 95% Bootstrap confidence interval of [0.0918, 0.2021] excluding zero, indicating a significant mediation effect. The indirect effect accounted for 27.4% of the total effect, while the direct effect accounted for 72.6%, representing a partial mediation. These results highly align with the path coefficients and mediation effect sizes obtained from the PLS-SEM baseline model, effectively verifying that the conclusions regarding the core mediation mechanism are robust and reliable.

**Table 11:** Mediation model test results.

Dependent variable	Predictor variable	*R* ^2^	*F*	*β*	SE	*t*
Anti-algorithm behavior	Perceived algorithmic control	0.2702	178.1177	0.5116	0.0383	13.3461^***^
Perceived overqualification	Perceived algorithmic control	0.3537	131.3505	0.3878	0.0423	9.1669^***^
Perceived overqualification	Anti-algorithm behavior			0.2860	0.0430	6.6534^***^
Perceived algorithmic control	Perceived overqualification	0.2941	200.4065	0.5342	0.0377	14.1565^***^

*p < 0.05, **p < 0.01, ***p < 0.001.

**Table 12:** Mediation effect test results.

Effect type	Effect estimate	SE	*T*	*p*	Bootstrap95%CI		Effect proportion (%)
					Lower	Upper	
Total effect	0.5342	0.0377	14.1565	0.000			
Direct effect	0.3878	0.0423	9.1669	0.000			72.6
Mediation effect	0.1463	0.0282	-	-	0.0918	0.2021	27.4

*p < 0.05, **p < 0.01, ***p < 0.001.

#### 4.3.7 Control variable robustness check

All control variables in this study were uniformly incorporated into the model using dummy coding. The reference group settings were completely consistent with the baseline categories automatically excluded in SPSS analysis, ensuring full reproducibility. Gender, employment type, recruitment channel, and located city were nominal categorical variables. For gender, males (corresponding to code 1.0) served as the reference group, and females (corresponding to code 2.0) were included as a dummy variable; for employment type, full-time riders (corresponding to code 1.0) were the reference group, and part-time riders (corresponding to code 2.0) were included as a dummy variable; for recruitment channel, offline station recruitment was the reference group, and online community recruitment was included as a dummy variable; for located city, Hangzhou was the reference group, and Shanghai and Suzhou were included as dummy variables. Age, education, and years of experience were ordinal categorical variables. To avoid estimation bias caused by forced equidistant assignment, they were also treated with dummy coding. For age, the 40–49 years group (corresponding to code 4.0) was the reference group, and the groups of 20 years and under, 21–29 years, 30–39 years, and 50 years and above were included as dummy variables; for education, the associate degree group (corresponding to code 3.0) was the reference group, and the groups of junior high school and below, high school/secondary vocational school, bachelor’s degree, and graduate degree and above were included as dummy variables; for years of experience, the 4–6 years group (corresponding to code 2.0) was the reference group, and the groups of 3 years and below, 7–9 years, and 10 years and above were included as dummy variables.

To rule out the confounding interference of demographic characteristics, regional differences, and recruitment channels on the core conclusions, this study incorporated a total of 7 control variables—gender, age, education, employment type, years of experience, located city, and recruitment channel—into the PLS-SEM model in the form of dummy coding for re-estimation. The results are shown in Table 13.

**Table 13:** Path coefficients and their significance.

Paths	Estimated value	Standard deviation	*T*	2.50%	97.50%
gender_2 → Anti-algorithm behavior	−0.067	0.04	1.678	−0.148	0.008
edu_4 → Anti-algorithm behavior	−0.039	0.037	1.033	−0.114	0.032
city_1 → Anti-algorithm behavior	0.035	0.039	0.896	−0.046	0.11
edu_5 → Anti-algorithm behavior	−0.046	0.03	1.563	−0.111	0.007
working years_1 → Anti-algorithm behavior	−0.078	0.049	1.604	−0.178	0.013
working years_3 → Anti-algorithm behavior	0.037	0.042	0.886	−0.044	0.122
working years_4 → Anti-algorithm behavior	0.066	0.033	1.992	0.001	0.132
city_3 → Anti-algorithm behavior	0.051	0.039	1.299	−0.026	0.128
employment type_2 → Anti-algorithm behavior	0.058	0.039	1.487	−0.018	0.134
recruitment channel_1 → Anti-algorithm behavior	−0.003	0.035	0.093	−0.073	0.063
age_1 → Anti-algorithm behavior	−0.034	0.039	0.853	−0.111	0.042
age_2 → Anti-algorithm behavior	−0.001	0.044	0.029	−0.089	0.084
age_3 → Anti-algorithm behavior	0.031	0.048	0.634	−0.066	0.125
age_5 → Anti-algorithm behavior	0.09	0.038	2.368	0.014	0.161
edu_1 → Anti-algorithm behavior	−0.044	0.041	1.092	−0.128	0.032
edu_2 → Anti-Anti-algorithm behavior	−0.051	0.043	1.18	−0.136	0.035
Perceived algorithmic control → Anti-algorithm behavior	0.407	0.048	8.533	0.312	0.498
Perceived algorithmic control → Perceived overqualification	0.533	0.043	12.261	0.442	0.611
Perceived overqualification → Anti-algorithm behavior	0.275	0.048	5.736	0.18	0.368
Perceived algorithmic control → Perceived overqualification → Anti-algorithm behavior	0.147	0.028	5.291	0.095	0.205
Perceived algorithmic control → Anti-algorithm behavior	0.554	0.048	11.451	0.447	0.640
Perceived algorithmic control → Perceived overqualification	0.533	0.043	12.261	0.442	0.611
Perceived overqualification → Anti-algorithm behavior	0.275	0.048	5.736	0.18	0.368

*p < 0.05, **p < 0.01, ***p < 0.001.

After including the control variables, the core mechanism was highly consistent with the baseline model conclusions: the direct effect of perceived algorithmic control on anti-algorithm behaviors was significantly positive (*β* = 0.407, *p* < 0.001); perceived algorithmic control positively predicted perceived overqualification (*β* = 0.533, *p* < 0.001); and perceived overqualification positively predicted anti-algorithm behaviors (*β* = 0.275, *p* < 0.001). The mediating effect of perceived overqualification was 0.147, corresponding to a *T*-value of 5.291 (*p* < 0.001), indicating a significant mediation effect. The total effect of perceived algorithmic control on anti-algorithm behavior was 0.554 (*p* < 0.001), with the mediating effect accounting for 26.5% of the total effect, which remained a partial mediation pattern.

Compared with the baseline model without control variables, the coefficient fluctuations of the three core paths were all less than 2%, and the mediating effect value differed by only 0.003. The direction of the effects and statistical significance remained completely unchanged, indicating that demographic characteristics, regional differences, and recruitment channels do not exert a substantial interference on the core mediating mechanism of this study. The research conclusions possess good robustness.

The effects of the control variables showed that only the 50 years and above age group (*β* = 0.090, *p* = 0.018) and the 10 years and above experience group (*β* = 0.066, *p* = 0.046) had a weakly significant positive effect on anti-algorithm behaviors. The effects of other control variables, such as gender, education, employment type, located city, and recruitment channel, did not reach statistical significance, further verifying the independence of the core mechanism.

## 5 Conclusion

### 5.1 Research findings

Grounded in Conservation of Resources Theory, this study examines how perceived algorithmic control relates to anti-algorithm behavior among gig workers, with particular attention to the mediating role of perceived overqualification. Drawing on a three-wave longitudinal design, four key findings emerge.

First, perceived algorithmic control is positively associated with anti-algorithm behavior (*β* = 0.400, *p* < 0.001). Algorithmic systems—through rigid normative guidance, real-time monitoring, and behavioral constraints—are positively associated with perceived overqualification, thereby relating to anti-algorithm behavior aimed at reclaiming agency. Second, perceived algorithmic control is positively associated with perceived overqualification (*β* = 0.533, *p* < 0.001). In tightly governed algorithmic environments, standardized evaluation criteria often fail to recognize workers’ full skill sets, intensifying the perception of person-job mismatch.

Third, perceived overqualification is positively associated with anti-algorithm behavior (*β* = 0.282, *p* < 0.001). Workers who perceive their competencies as underutilized are more likely to seek alternative means of redress when conventional channels prove insufficient, with anti-algorithm behavior serving as a form of behavioral dissent.

Fourth, perceived overqualification significantly mediates the relationship between perceived algorithmic control and anti-algorithm behavior (indirect effect *β* = 0.150, *p* < 0.001). This indicates that perceived algorithmic control is associated with behavioral outcomes not only directly but also indirectly—by being associated with heightened perceived overqualification—suggesting a critical psychological pathway linking algorithmic governance to employee resistance.

### 5.2 Theoretical contributions

This study advances the literature in three substantive ways.

First, by integrating Conservation of Resources Theory (Hobfoll, 1989), we offer a novel theoretical lens for understanding the perceived algorithmic control–anti-algorithm behavior nexus. While COR theory has been widely applied to explain resource loss and defensive behavior in organizational settings, its application to algorithmic management remains underexplored. We demonstrate that algorithmic control—through rigid normative guidance, real-time monitoring, and behavioral constraints—depletes workers’ key resources (e.g., autonomy, temporal flexibility, and decision-making authority), thereby triggering defensive responses such as anti-algorithm behavior. This extends COR theory into the algorithmic management domain and deepens our understanding of the specific resource-loss mechanisms that motivate counter-algorithmic actions.

Second, we extend the perceived overqualification literature beyond its traditional organizational context. Prior research has largely examined overqualification in relation to job mismatch, limited career advancement, or person-organization fit (Khan et al., 2022). We reposition perceived overqualification within the gig economy by showing how algorithmic management practices—including standardized task allocation, opaque performance evaluation, and automated reward–punishment systems—systematically generate feelings of skill underutilization. This reconceptualization broadens the theoretical boundaries of overqualification research and offers a platform-specific mechanism that complements existing accounts.

Third, we construct an integrative theoretical model—perceived algorithmic control → perceived overqualification → anti-algorithm behavior—that unifies these constructs within a single analytical framework. Unlike prior studies that examine these relationships in isolation, model delineates the temporal predictive pathways linking algorithmic governance to employee resistance. This not only enhances theoretical explanatory power but also provides an empirically grounded basis for designing interventions that mitigate anti-algorithm behavior in platform economies.

### 5.3 Practical implications

This study provides differentiated decision-making references for platform managers, policymakers, and organizational practitioners to optimize algorithmic governance and guide rider behavior in the gig economy context. It is particularly important to note that anti-algorithm behaviors exhibit significant heterogeneity: they encompass both strategic workarounds and the expression of reasonable demands adopted by riders to adapt to real-world work scenarios, as well as deceptive and potentially harmful violations involving false status reporting and location tampering. These two categories require distinct policy approaches and should not be treated with a one-size-fits-all approach.

First, strong algorithmic control—relying on rigid task allocation, ambiguous performance evaluation, and automated reward and penalty systems—is associated with riders’ perception of skill underutilization and psychological pressure, thereby relating to various coping behaviors. Therefore, platform operators should move beyond the singular management logic of “efficiency first” and incorporate workers’ psychological perceptions and person-job fit experiences into the scope of algorithmic governance considerations. Recognizing the value of riders’ diverse skills in algorithm design and establishing open, transparent evaluation and reward/penalty rules can help alleviate riders’ perceived overqualification, thereby reducing the emergence of passive coping behaviors at their source.

Second, autonomy loss is the core pathway associated with riders’ defensive coping. Ensuring workers’ moderate work autonomy is not merely an ethical requirement but a crucial lever for reducing non-compliant coping behaviors and enhancing employment stability. Platforms can embed autonomy-supportive designs into their management systems, such as flexible scheduling mechanisms, a certain degree of autonomous order selection, and the explainability of algorithmic decision rules. Such optimizations can reduce riders’ perception of skill underutilization and being controlled, thereby decreasing the probability of various anti-algorithm behaviors.

Third, regarding heterogeneous anti-algorithm behavior, a governance strategy that “differentiates the nature of the behavior and combines guidance with restriction” should be adopted, avoiding a solely punishment-oriented governance logic. On the one hand, regarding constructive workarounds adopted by riders due to real-world contextual constraints (e.g., adjusting delivery operations with consumer consent, selecting reasonable order-accepting strategies) and the expression of reasonable demands, platforms should not simply prohibit them. Instead, these should be used as a reference for algorithmic optimization and absorbed into the iteration of algorithmic rules to enhance the algorithm’s contextual adaptability. On the other hand, for deceptive behaviors that may harm the rights and interests of consumers and merchants or pose service safety hazards—such as reporting false information and tampering with locations—platforms must still regulate and constrain them through refined rules and technical means, safeguarding the bottom line of service quality and safety. On this basis, platforms can establish complementary psychological support and demand feedback systems to guide riders in expressing their demands through formal channels, thereby substituting non-compliant coping behaviors.

Fourth, establishing a normalized two-way communication mechanism between the platform and gig workers is the critical foundation for accurately identifying workers’ demands and resolving conflicts in a categorized manner. Relying on real-time rider feedback channels, platforms can promptly distinguish reasonable work demands from motives for non-compliant behaviors, and optimize algorithmic rules and management mechanisms accordingly: responding and iterating on reasonable demands in a timely manner, while accurately guiding and regulating non-compliant behaviors. This approach not only improves riders’ work experience and retains high-skilled workers but also maintains platform service order and multi-party rights and interests, ultimately supporting the long-term sustainable development of the platform.

## 6 Limitations and future directions

While this study offers both theoretical and empirical contributions, several limitations warrant attention and suggest avenues for future research.

First, the sample was restricted to food delivery workers, limiting generalizability across gig economy sectors. Workers in ride-hailing, online tutoring, or freelance platforms face distinct forms of algorithmic control—such as dispatch fairness or pedagogical interference—that may shape perceived overqualification and anti-algorithm behavior differently. Future research should adopt stratified sampling across multiple gig sectors to examine sector-specific moderators and enhance external validity.

Second, the study is situated within the Chinese cultural context, where collectivist values may attenuate resistance behaviors relative to individualist settings (Hofstede, 2001). Cross-cultural comparisons remain absent, constraining the universality of the proposed model. Future work should incorporate multi-country designs to test whether cultural dimensions—particularly power distance and individualism–collectivism—moderate the hypothesized pathways.

Third, although the three-wave longitudinal design strengthens temporal ordering, it cannot fully rule out unmeasured confounders or reverse causality. The one-week interval was chosen to balance recall accuracy against attrition risk, yet this trade-off leaves residual endogeneity concerns. Furthermore, only one measure of each construct was collected, and baseline levels of the mediator and outcome were not controlled. Therefore, unobserved confounding, stable individual differences, and reverse relationships remain possible. Future studies could employ experimental or quasi-experimental designs, or extend the temporal lag between waves, to establish more robust causal inference.

Fourth, anti-algorithm behavior constitutes a sensitive and potentially stigmatized conduct, raising concerns about social desirability bias in self-reported measures. Although full anonymity and confidentiality assurances were provided to mitigate underreporting, this threat cannot be entirely eliminated. Integrating objective behavioral data—such as platform logs or digital trace data—would complement self-reports and strengthen the validity of future findings.

Regarding model fit indices, the Normed Fit Index (NFI) in this study was 0.794, falling short of the traditional reference threshold of 0.90. In the evaluation framework of partial least squares structural equation modeling (PLS-SEM), goodness-of-fit indices serve merely as an auxiliary reference dimension for model quality. The core criteria for assessment are the reliability and validity of the measurement model, the theoretical consistency and statistical significance of the structural paths, and the model’s explanatory power and predictive relevance. In conjunction with the preceding test results, the reliability and validity of all latent variables in this study conform to academic standards. Furthermore, the core hypothetical paths are all significant and consistent with the theoretical logic, and both the model’s explanatory power and predictive relevance meet acceptable standards. Therefore, the overall model quality and research conclusions are reliable. Future research may further optimize model fit performance by incorporating boundary condition variables and expanding the sample size.

## Data Availability

The original contributions presented in the study are included in the article/Supplementary material, further inquiries can be directed to the corresponding author.
