# Supplementary material for: Perceived algorithmic control and anti-algorithm behaviors: the catalytic role of perceived overqualification
Source: Front Psychol. 2026 Sep 10;17:1774556. doi: 10.3389/fpsyg.2026.1774556 (PMC13600867; doi:10.3389/fpsyg.2026.1774556)
Supplement: Supplementary file 1 [file Data_Sheet_2.pdf]

crosstab

Case Handling Summary

	individual cases					
	Valid		Missing		Total	
	N	Percentage	N	Percentage	N	Percentage
Gender * Lost to follow-up	508	100.0%	0	0.0%	508	100.0%

Gender ★ Is the User Lost? Cross-Tabulation

count

		exit status: No	yes	Total
Sex	Man	309	20	329
	Woman	174	5	179
Total		483	25	508

Chi-Square Test

	Price	Freedom degree	Progressive significance (two-tailed)	Exact Significance (Two-tailed)	Exact Significance (One-Tailed)
Pearson's chi-square statistic	2.675[a]	1	.102		
Continuous adjustment b	2.019	1	.155		
Likelihood ratio	2.919	1	.088		
Fisher's exact test				.133	.074
Linear correlation	2.670	1	.102		
Number of valid cases	508				

a. The expected count for 0 cells (.0%) is less than 5; the minimum expected count is 8.81.

b. Calculations are performed only for a 2x2 table.

Symmetric Measurement

		Price	Asymptotic standard error a	Approximation Tb	Progressive significance
From nominal to nominal	Coefficient of contingency	.072			.102
Interval to Interval	Pearson R	-.073	.038	-1.637	.102[c]
From ordered to ordered	Spearman's correlation coefficient	-.073	.038	-1.637	.102[c]
Number of valid cases		508			

a. The null hypothesis is not assumed.

b. Use the asymptotic standard error under the null hypothesis.

c. Based on the normal approximation.

Crosstab

Case Handling Summary

	individual cases					
	Valid		Missing		Total	
	N	Percentage	N	Percentage	N	Percentage
Age * Whether the participant has dropped out	508	100.0%	0	0.0%	508	100.0%
Educational attainment * Whether the student has dropped out	508	100.0%	0	0.0%	508	100.0%
Work arrangement * Employee turnover?	508	100.0%	0	0.0%	508	100.0%
Years of service * Whether the employee has left the company	508	100.0%	0	0.0%	508	100.0%

Age ★ Is the user lost?

Crosstab

count

		exit status: No	yes	Total
Age	Up to 20 years of age	50	4	54
	21-29	125	6	131
	30-39	115	5	120
	40-49	159	9	168
	Age 50 or older	34	1	35
Total		483	25	508

Chi-Square Test

	Price	Freedom degree	Progressive significance (two-tailed)
Pearson's chi-square statistic	1.279[a]	4	.865
Likelihood ratio	1.248	4	.870
Linear correlation	.284	1	.594
Number of valid cases	508		

a. The expected count for 2 cells (20.0%) is less than 5; the minimum expected count is 1.72.

Symmetric Measurement

		Price	Asymptotic standard error [a]	Approximation Tb	Progressive significance
From nominal to nominal	Coefficient of contingency	.050			.865
Interval to Interval	Pearson R	-.024	.046	-.532	.595[c]
From ordered to ordered	Spearman's correlation coefficient	-.020	.046	-.446	.656[c]
Number of valid cases		508			

a. The null hypothesis is not assumed.

b. Use the asymptotic standard error under the null hypothesis.

c. Based on the normal approximation.

Educational attainment ★ Has the student dropped out?

Crosstab

count

		exit status: No	yes	Total
Educational status	Junior high school or below	71	3	74
	Senior middle school	166	11	177
	Specialized Field	195	10	205
	Undergraduate course	37	1	38
	Postgraduate degree or above	14	0	14
Total		483	25	508

Chi-Square Test

	Price	Freedom degree	Progressive significance (two-tailed)
Pearson's chi-square statistic	1.903[a]	4	.754
Likelihood ratio	2.635	4	.621
Linear correlation	.519	1	.471
Number of valid cases	508		

a. The expected count for 3 cells (30.0%) is less than 5; the minimum expected count is 0.69.

Symmetric Measurement

		Price	Asymptotic standard error [a]	Approximation Tb	Progressive significance
From nominal to nominal	Coefficient of contingency	.061			.754
Interval to Interval	Pearson R	-.032	.036	-.720	.472[c]
From ordered to ordered	Spearman's correlation coefficient	-.029	.040	-.654	.513[c]
Number of valid cases		508			

a. The null hypothesis is not assumed.

b. Use the asymptotic standard error under the null hypothesis.

c. Based on the normal approximation.

Work arrangement ★ Employee turnover?

Crosstab

count

		exit status: No	yes	Total
Work Format	Hold two or more posts concurrently	237	13	250
	Full-time	246	12	258
Total		483	25	508

Chi-Square Test

	Price	Freedom degree	Progressive significance (two-tailed)	Exact Significance (Two-tailed)	Exact Significance (One-Tailed)
Pearson's chi-square statistic	.082[a]	1	.775		
Continuous adjustment b	.007	1	.936		
Likelihood ratio	.082	1	.775		
Fisher's exact test				.839	.468
Linear correlation	.082	1	.775		
Number of valid cases	508				

a. The expected count for 0 cells (.0%) is less than 5; the minimum expected count is 12.30.

b. Calculations are performed only for a 2x2 table.

Symmetric Measurement

		Price	Asymptotic standard error [a]	Approximation Tb	Progressive significance
From nominal to nominal	Coefficient of contingency	.013			.775
Interval to Interval	Pearson R	-.013	.044	-.285	.775[c]
From ordered to ordered	Spearman's correlation coefficient	-.013	.044	-.285	.775[c]
Number of valid cases		508			

a. The null hypothesis is not assumed.

b. Use the asymptotic standard error under the null hypothesis.

c. Based on the normal approximation.

Years of service ★ Has the employee left the company?

Crosstab

count

		exit status: No	yes	Total
Working life	3 years or less	60	2	62
	4-6	197	9	206
	7-9	196	11	207
	10 years or more	30	3	33
Total		483	25	508

Chi-Square Test

	Price	Freedom degree	Progressive significance (two-tailed)
Pearson's chi-square statistic	1.810[a]	3	.613
Likelihood ratio	1.629	3	.653
Linear correlation	1.450	1	.228
Number of valid cases	508		

a. For 2 cells (25.0%), the expected count is less than 5; the minimum expected count is 1.62. The correlation measure was not calculated for the cross-tabulation involving the recruitment channel * whether the subject was lost. In each two-way table, at least

Symmetric measurement

		Price	Asymptotic standard error [a]	Approximation Tb	Progressive significance
From nominal to nominal	Coefficient of contingency	.060			.613
Interval to Interval	Pearson R	.053	.045	1.205	.229[c]
From ordered to ordered	Spearman's correlation coefficient	.051	.045	1.140	.255[c]
Number of valid cases		508			

a. The null hypothesis is not assumed.

b. Use the asymptotic standard error under the null hypothesis.

c. Based on the normal approximation.

crosstab

Warn

A cross-tabulation is not used to calculate the correlation measure for whether the city in question has been lost. In each two-way table, at least one of the variables on which the correlation measure is based is a constant.
one of the variables on which the correlation measure is based is a constant.

Case Handling Summary

	individual cases					
	Valid		Missing		Total	
	N	Percentage	N	Percentage	N	Percentage
City * – Has the city been lost?	483	95.1%	25	4.9%	508	100.0%
Recruitment channels * Customer churn?	483	95.1%	25	4.9%	508	100.0%

City ★ Has the city been lost?

Crosstab

count

		exit status: No	Total
City	Shanghai	142	142
	Hangzhou	250	250
	Suzhou	91	91
Total		483	483

Chi-Square Test

	price
Pearson's chi-square statistic	.[a]
Number of valid cases	483

a. Since the "exit status" variable is a constant, no statistical analysis was performed.

Symmetric Measurement

		price
From nominal to nominal	Coefficient of contingency	.[a]
Number of valid cases		483

a. Since the "exit status" variable is a constant, no statistical analysis was performed.

Recruitment Channels ★ Customer Churn?

Crosstab

count

		exit status: No	Total
Recruitment Channels	Offline	102	102
	On-line	381	381
Total		483	483

Chi-Square Test

	price
Pearson's chi-square statistic	.[a]
Number of valid cases	483

a. Since the "exit status" variable is a constant, no statistical analysis was performed.

Symmetric Measurement

		price
From nominal to nominal	Coefficient of contingency	.[a]
Number of valid cases		483

a. Since the "exit status" variable is a constant, no statistical analysis was performed.

T-checkout

Group Statistics

	Has the user churned?	Number of cases	Average value	Standard error	Standard Error Mean
pac	Deny	483	3.8882	.81983	.03730
	Yes	25	3.8600	.74330	.14866

Independent Samples Test

		Levin's variance homogeneity test		Mean Equality t-Test			
		F	Conspicuousness	t	Freedom degree	Significance (two-tailed)	Mean Difference
pac	Assume equal variances	.619	.432	.168	506	.866	.02820
	Does not assume equal variance			.184	27.112	.855	.02820

Independent Samples Test

	Mean Equality T-Test		
		Difference – 95% Confidence Interval	
	Standard Error Difference	Lower limit	Upper bound
PAC – Assuming equal variance	.16744	-.30077	.35717
Does not assume equal variance	.15327	-.28622	.34262

Independent Samples Effect Size

		Standardized measure a	Point estimation	95% confidence interval	
				Lower limit	Upper bound
pac	Cohen d	.81636	.035	-.367	.437
	Hedges Adjustment	.81758	.034	-.367	.436
	Glass Delta	.74330	.038	-.365	.440

a. The denominator used when estimating the effect size.
Cohen's d uses the pooled standard deviation.
Hedges' method uses the aggregated standard deviation, supplemented by a correction factor.
Glass Delta uses the sample standard deviation of the control group.

Matrix

Run MATRIX procedure:

PROCESS Procedure for SPSS Version 4.2

Written by Andrew F. Hayes, Ph.D. www.afhayes.com

Documentation available in Hayes (2022). www.guilford.com/p/hayes3

Model: 4

Y : aab

X : poq
M : pac

Sample
Size: 483

****
OUTCOME VARIABLE:
pac

Model Summary

R	R-sq	MSE	F	df1	df2	p
.5198	.2702	.4915	178.1177	1.0000	481.0000	.0000

Model

	coeff	se	t	p	LLCI	ULCI
constant	1.8151	.1586	11.4468	.0000	1.5036	2.1267
poq	.5282	.0396	13.3461	.0000	.4505	.6060

Standardized coefficients

	coeff
poq	.5198

****
OUTCOME VARIABLE:
aab

Model Summary

R	R-sq	MSE	F	df1	df2	p
.5947	.3537	.4232	131.3505	2.0000	480.0000	.0000

Model

	coeff	se	t	p	LLCI	ULCI
constant	1.3855	.1660	8.3473	.0000	1.0593	1.7116
poq	.2860	.0430	6.6534	.0000	.2016	.3705
pac	.3878	.0423	9.1669	.0000	.3047	.4710

Standardized coefficients

	coeff
poq	.2858
pac	.3938

************************** TOTAL EFFECT MODEL ****************************
OUTCOME VARIABLE:
aab

Model Summary

R	R-sq	MSE	F	df1	df2	p
.4905	.2406	.4963	152.3678	1.0000	481.0000	.0000

Model

	coeff	se	t	p	LLCI	ULCI
constant	2.0895	.1593	13.1135	.0000	1.7764	2.4025

poq	.4909	.0398	12.3437	.0000	.4128	.5691

Standardized coefficients

	coeff
poq	.4905

************** TOTAL, DIRECT, AND INDIRECT EFFECTS OF X ON Y **************

Total effect of X on Y

Effect	se	t	p	LLCI	ULCI	c_cs
.4909	.0398	12.3437	.0000	.4128	.5691	.4905

Direct effect of X on Y

Effect	se	t	p	LLCI	ULCI	c'_cs
.2860	.0430	6.6534	.0000	.2016	.3705	.2858

Indirect effect(s) of X on Y:

	Effect	BootSE	BootLLCI	BootULCI
pac	.2049	.0376	.1348	.2810

Completely standardized indirect effect(s) of X on Y:

	Effect	BootSE	BootLLCI	BootULCI
pac	.2047	.0356	.1377	.2770

*********************** ANALYSIS NOTES AND ERRORS ************************

Level of confidence for all confidence intervals in output:
95.0000

Number of bootstrap samples for percentile bootstrap confidence intervals:
5000

------ END MATRIX -----

Factor Analysis

[Dataset 1] C:\Users\lichaoyang\Desktop\20260708.sav

Dependency Matrix

		PAC1	PAC2	PAC3	PAC4	POQ1	POQ2	POQ3
Relativity	PAC1	1.000	.539	.592	.538	.296	.437	.379
	PAC2	.539	1.000	.510	.492	.265	.414	.300
	PAC3	.592	.510	1.000	.454	.156	.284	.334
	PAC4	.538	.492	.454	1.000	.369	.276	.376
	POQ1	.296	.265	.156	.369	1.000	.444	.361
	POQ2	.437	.414	.284	.276	.444	1.000	.325
	POQ3	.379	.300	.334	.376	.361	.325	1.000
	POQ4	.348	.273	.205	.257	.461	.499	.392
	AAB1	.311	.385	.269	.421	.314	.237	.285
	AAB2	.304	.346	.191	.374	.368	.314	.272
	AAB3	.425	.381	.299	.281	.248	.355	.329
	AAB4	.415	.391	.241	.426	.267	.328	.320

Dependency Matrix

		POQ4	AAB1	AAB2	AAB3	AAB4
Correlation	PAC1	.348	.311	.304	.425	.415
	PAC2	.273	.385	.346	.381	.391
	PAC3	.205	.269	.191	.299	.241
	PAC4	.257	.421	.374	.281	.426
	POQ1	.461	.314	.368	.248	.267
	POQ2	.499	.237	.314	.355	.328
	POQ3	.392	.285	.272	.329	.320
	POQ4	1.000	.234	.215	.308	.255
	AAB1	.234	1.000	.580	.470	.539
	AAB2	.215	.580	1.000	.401	.480
	AAB3	.308	.470	.401	1.000	.539
	AAB4	.255	.539	.480	.539	1.000

KMO and Bartlett's Test

KMO Sample Adequacy Measure.		.878
Bartlett's sphericity test	Approximate chi-square statistic	2142.749
	Freedom degree	66
	conspicuousness	.000

Common Factor Variance

	Initial	Extract
PAC1	1.000	.712
PAC2	1.000	.606
PAC3	1.000	.747
PAC4	1.000	.548
POQ1	1.000	.621
POQ2	1.000	.596
POQ3	1.000	.422
POQ4	1.000	.692
AAB1	1.000	.710
AAB2	1.000	.655
AAB3	1.000	.504
AAB4	1.000	.641

Extraction method: Principal Component Analysis (PCA).

Total Variance Explanation

	Initial eigenvalue			Extract the sum of squares of the loads			Rotational
Ingredient	Total	Variance percentage	Accumulate%	Total	Variance percentage	Accumula-te%	load... Total
1	4.975	41.461	41.461	4.975	41.461	41.461	2.611
2	1.254	10.450	51.912	1.254	10.450	51.912	2.574
3	1.224	10.199	62.111	1.224	10.199	62.111	2.269
4	.798	6.653	68.764				
5	.720	5.998	74.762				
6	.536	4.464	79.226				
7	.501	4.175	83.401				
8	.477	3.975	87.376				
9	.448	3.737	91.112				
10	.389	3.244	94.356				
11	.356	2.964	97.320				
12	.322	2.680	100.000				

Total Variance Explanation

Ingredient	Rotational load squared sum Variance percentage	Accumulate%
1	21.758	21.758
2	21.448	43.206
3	18.905	62.111
4		
5		
6		
7		
8		
9		
10		
11		
12		

Extraction method: Principal Component Analysis (PCA).

Component Matrix [a]

	ingredient 1	2	3
PAC1	.735		
PAC2	.697		
PAC4	.691		
AAB4	.684		
AAB1	.659	-.523	
AAB3	.657		
POQ2	.630		
AAB2	.629		
POQ3	.596		
PAC3	.590		-.564
POQ1	.575		.506
POQ4	.559		

Extraction method: Principal Component Analysis (PCA).

a. Three components were extracted.

Rotated Component Matrix [a]

	ingredient		
	1	2	3
AAB1	.816		
AAB2	.778		
AAB4	.743		
AAB3	.611		
PAC3		.859	
PAC1		.766	
PAC2		.691	
PAC4		.613	
POQ4			.817
POQ1			.740
POQ2			.704
POQ3			.514

Extraction method: Principal Component Analysis (PCA).
Rotation method: Caesar normalization maximum variance method.a
a. The rotation has converged after 5 iterations.

Component Transformation Matrix

Ingredient	1	2	3
1	.604	.599	.526
2	-.786	.338	.517
3	.132	-.726	.675

Extraction method: Principal Component Analysis (PCA).
Rotation method: Caesar normalization with maximum variance method.
